# Numerical investigation on characteristics of vortex dissipation in multi-horizontal submerged jets stilling basin

**DOI:** 10.1371/journal.pone.0301423

**Published:** 2024-05-23

**Authors:** Ruichang Hu, Lan Yang, Hao Yuan, Chunhua Xia, Qian Sun, Lei Jiang, Wei Diao, Yunfei Mao

**Affiliations:** 1 Southwest Research Institute for Hydraulic and Water Transport Engineering, Chongqing Jiaotong University, Chongqing, China; 2 School of River and Ocean Engineering, Chongqing Jiaotong University, Chongqing, China; 3 School of Civil Engineering, Chongqing Jiaotong University, Chongqing, China; 4 Power China Kunming Engineering Corporation Limited, Kunming, China; 5 Chongqing Xike Water Transport Engineering Consulting Co., LTD, Chongqing Jiaotong University, Chongqing, China; KTH Royal Institute of Technology: Kungliga Tekniska Hogskolan, SWEDEN

## Abstract

Multi-horizontal submerged jets stilling basins have been utilized in large-scale water conservancy and hydropower projects due to its stable flow pattern, high energy dissipation rate and less atomization. This study employs vorticity criterion, Q criterion, λ_2_ criterion and Ω criterion to investigate the characteristics of vortex formation and turbulent dissipation in multi-horizontal submerged jets stilling basins with various configurations, including crest overflowing orifice alone (COO), combination of crest overflowing orifice and mid-discharge orifice (COO-MO) and mid-discharge orifice alone (MO). The results indicate that the Q criterion and λ_2_ criterion are effective in identifying vortex structure within multi-horizontal submerged jets stilling basin. Specifically, the stronger intensity of vortex structure and vortex dissipation are mainly distributed in the vicinity of the vertical drop, which gradually weakens for the increasing distance to the vertical drop. Furthermore, the intensity and number of vortexes with COO-MO are the largest. This conclusion can provide guidance for energy dissipation and bottom protection of stilling pool.

## Introduction

The energy dissipation of water with a high-water level and large flow rate has always been the focus of the research in water conservancy and hydropower projects. A single submerged jet will easily cause excessive impact damage to the bottom of the stilling basin while dissipating energy in the water body, but multiple jets can effectively avoid this problem [1]. A type of stilling basin with multi-horizontal submerged jets was proposed by Deng et al. [2], which had a weak atomization and a steady flow pattern. At the same time, the vortex born between different jets could increase the energy dissipation rate and reduce the impact on the ecological environment.

The investigation on stilling basin with multi-horizontal submerged jets have been conducted by numerous scholars [2–6]. Zhang et al. [7] established the relationship between the energy dissipation rate and the ratio of jet outlet width to the width of the stilling basin, and the water depth in the multi-horizontal submerged jets stilling basin. And they recommended that the velocity of outlet of the gutter and in the vicinity of slab, and the damage to the bottom slab were reduced, and the energy dissipation could be guaranteed simultaneously, when using multi-horizontal submerged jets. Li et al. [8] carried out various physical tests on the shape of the outlet of the orifice, and the space between crest overflowing orifice (COO) and mid-discharge orifice (MO). It was considered that the mesoporous groove is preferably of equal width or slight shrinkage and the extension of the pier was beneficial to stabilize the flow regime. Chen et al. [9] analyzed the velocity and water depth of multi-horizontal submerged jets stilling basin in different scale models through experiments and concluded that the time-averaged hydraulic characteristics in the stilling basin were less impacted by the scale. To analyze the three-dimensional structure of submerged jets and evaluate the influence of downstream submerged flow field, laboratory velocity measurements were carried out by Moradi et al. and the results show that the Reynolds shear stress and vorticity increase with the increase of jet depth [10]. Zhou et al. [11] conducted experimental and numerical studies on a new partial FGP scheme and found that the partial FGP scheme (the alternation of flaring and no flaring gate piers in chambers) can effectively suppress the inundated hydraulic jump and high-speed water jet in the upstream area of the stilling pool.

Although vortexes can increase energy dissipation in the water, however, which may cause some damage to the stilling basin when the vortex intensity is strong enough [12]. A large number of researchers conducted a lot of research on the vortex with dividing the vortex into vertical-axis vortex and horizontal-axis vortex in multi-horizontal submerged jets stilling basin. Gao et al. [13] found that vertical-axis vortexes existed in multi-horizontal submerged jets stilling basin using the gravel tracing method in the test, but the position of the vortex and whether it penetrated could not be determined. Numerical calculation was gradually being applied in jet research due to its advantages of more detailed analysis [14–17]. The RNG *k—ε* turbulence model was adopted by Yang et al. [18] to analyze the vortex characteristics of the stilling basin. It is found that return swirls were formed on the upper and lower sides of the jet, but no obvious stable vertical axis vortex was observed because the height was too high. Deng et al. [19] pointed out that the vertical-axis vortex appeared at the bottom of the stilling basin after the water flow entered the basin, and then it would be influenced by the horizontal-axis vortex as the water gradually stabilized. To further analyze the variation of vortex in the whole depth of the stilling basin, Chen et al. [20] employed particle image velocimetry (PIV) to study the vortex structure and found that the vortex, changing with time with undergoing merger-split-disappearance, had obvious three-dimensional characteristics. The vertical-axis vortex was observed at the bottom of the stilling basin and the range, and the position of the core changed significantly with the increasing water depth. Zhang et al. [21] found that there were obvious horizontal-axis vortexes, changing with different position, under high-speed jets and no obvious penetration phenomenon in the horizontal-axis due to the influence of vertical-axis vortex.

The energy dissipation and destruction in the stilling basin is mainly achieved by the strength of vortex, not just the range of vortex. Therefore, it is not sufficient to study only the range of vortex and we should analyze the energy dissipation and destruction characteristics of the vortex in the stilling basin in combination with the distribution of vortex intensity. Vortex identification criteria [22,23] are widely employed by investigators to study the structure and intensity of vortexes. Four vortex identification criteria are adopted and then an optimal method, being suited for this type of flow with high shear and high Reynolds number, is selected to conduct an in-depth study of the vortex intensity distribution characteristics in multi-horizontal submerged jets stilling basin with various aerial drainage patterns.

## Model layout

### Model introduction

The present work is based on the prototype of xiangjiaba water conservancy project’s multi-horizontal submerged jets stilling basin. Numerous resources need to be consumed in the physical test with the actual model, thus a certain scale used in the physical test is necessary. In this study, the numerical models are established in the scale of 1:80 and the feasibility and accuracy of the numerical models are verified by the experimental data of the physical model. Then, the research on the vortex characteristics in multi-horizontal submerged jets stilling basin is performed.

**Fig 1:**
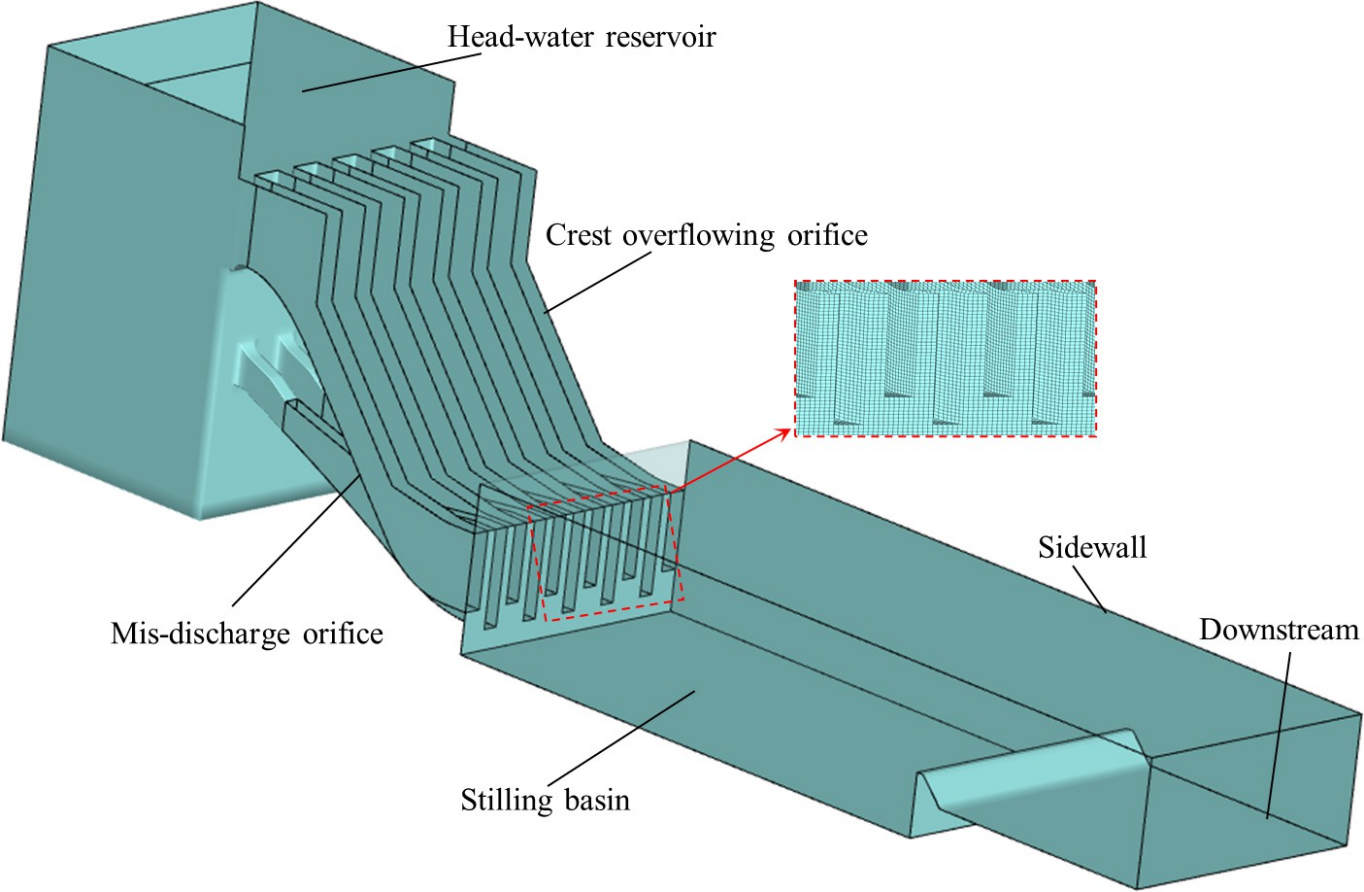
The layout of numerical model.

The model consists of the high-water reservoir, drainage orifice, stilling basin and downstream (shown in Fig 1). The drainage orifice consists of 6 crest overflowing orifices and 5 mid-discharge orifices, all of which were open overflow weir. In the actual model, the width of the COO and mid-discharge orifice is 8 m and 6 m respectively, all of which adopt horizontal drop. The length and wide of the stilling basin are 228 m and 108 m respectively and the height of tail ridge is 25m. The distance from the floor of COO and MO to the bottom of the basin is 16m and 8m, respectively. The flow rate of the COO, COO-MO and MO is 12800m³·s^-1^, 24900 m³·s^-1^, and 9560 m³·s^-1^, respectively.

In the present work, the scale model of 1:80 is adopted to analyze the vortex structure of the COO, COO-MO and MO, as shown in Table 1.

**Table 1:** Operating parameters in different cases.

series	scale	COO	MO	velocity (m·s^-1^)
case 1	1:80	6	0	0.11
case 2		6	5	0.20
case 3		0	5	0.08

### Governing equations

The high-speed water with strong anisotropy become very complicated when it quickly enters the stilling basin. Although direct numerical simulation (DNS) and large eddy simulation (LES) have obvious advantages in anisotropy, DNS and LES have high requirements regarding the grid resolution and are computationally expensive, while the Reynolds-Averaged Navier-Stokes (RANS) method is less resource intensive. At the same time, the model of this multi-horizontal submerged jets is generally large and belongs to the range of high Reynolds numbers, which will lead to the distortion of LES calculation. The renormalized group (RNG) *k—ε* turbulence model, proposed by Yakhot and Orszag (1986) [24], is adopted to carry out numerical calculations for that it has a well perform in the flow with a large degree of bending, and the corresponding turbulent kinetic energy (*k*) and dissipation rate (*ε*) control equations are shown as follows:

k equation:

$$\frac{\partial }{\partial t}(\rho k)+\frac{\partial }{\partial x_{i}}(\rho ku_{i})=\frac{\partial }{\partial x_{j}}\left[\alpha_{k}(\mu +\frac{\mu_{t}}{\sigma_{k}})\frac{\partial k}{\partial x_{j}}\right]+G_{k}-\rho \varepsilon$$ (1)

*ε* equation:

$$\frac{\partial }{\partial t}(\rho \varepsilon )+\frac{\partial }{\partial x_{i}}(\rho \varepsilon u_{i})=\frac{\partial }{\partial x_{j}}\left[\alpha_{\varepsilon }(\mu +\frac{\mu_{t}}{\sigma_{\varepsilon }})\frac{\partial \varepsilon }{\partial x_{i}}\right]+\frac{C_{1\varepsilon }^{*}\varepsilon }{k}G_{k}-C_{2\varepsilon }\rho \frac{\varepsilon^{2}}{k}$$ (2)

$$G_{k}=\mu_{t}\left(\frac{\partial u_{i}}{\partial x_{j}}+\frac{\partial u_{j}}{\partial x_{i}}\right)\frac{\partial u_{i}}{\partial x_{j}}$$ (3)

$$C_{1\varepsilon }^{*}=C_{1\varepsilon }-\frac{\eta (1-\eta /\eta_{0})}{1+\beta \eta^{3}}$$ (4)

$$\eta =\sqrt{\frac{G_{k}}{\rho C_{\mu }\varepsilon }}$$ (5)

$$\mu_{t}=\rho C_{\mu }\frac{k^{2}}{\varepsilon }$$ (6)

where, *μ* is the dynamic viscosity; *μ*_t_ is the turbulent viscosity; *ρ* is the corresponding density; *t* is time; and *C*_μ_, *C*_1ε_, *C*_2ε_, *β*, and *η*_0_ are the empirical constants. *C*_μ_ = 0.0845, *C*_1ε_ = 1.42, *C*_2ε_ = 1.68, *β* = 0.012, and *η*_0_ = 4.38 [25].

### Set and boundary conditions

The VOF model in the commercial software Fluent is utilized to calculate in the present work, which simulates immiscible fluids by solving separate momentum equations and processing the volume fraction of each fluid passing through the area [26]. The governing equations of the VOF model are presented in Eqs (7) and (9). The PISO algorithm proposed by Issa [27] is employed for the pressure-velocity coupling. The transient is selected in solver and differential viscosity model option is enabled in the RNG *k—ε* model. The structured grid and non-uniform grid are used in most locations of the model or locations where the grid is not well divided. The schematic diagrams of the local grids of the outlet of the orifice and overflow weir are shown in Fig 1.


$$\frac{\partial F}{\partial t}+u_{i}\frac{\partial F}{\partial x_{i}}=0$$ (7)

$$\rho =F_{w}\rho_{w}+\left(1-F_{w}\right)\rho_{a}$$ (8)

$$\mu =F_{w}\mu_{w}+\left(1-F_{w}\right)\mu_{a}$$ (9)

The boundary conditions are as follows:

1. Inlet boundary: A pressure-inlet is adopted for the water surface and standard atmospheric pressure is set in air-water interface.
2. Outlet boundary: A pressure-outlet is employed for the outlet of the numerical model.
3. Wall boundary: no-slip velocity boundary condition; The near-wall regions of the flow are analyzed using the standard wall function method.

### Model validation

The water in the stilling pool rolls violently, resulting an intensified anisotropy, when it quickly enters the stilling pool. Thus, it is important to verify the accuracy of turbulence model in multi-horizontal submerged jets in the stilling basin. The RNG *k—ε* turbulence model reflects the influence of small-scale vortices through large-scale motion and modified viscosity term, which makes the model more applicable to anisotropy. Please refer to reference [4] for the accuracy analysis of RNG *k—ε* turbulence model in a multi-layer horizontal submerged jet stilling pool.

## Vortex identification criterion theory

### Vorticity criterion

Vorticity criterion was proposed by Metcalfe et al. [28] in 1985, which calculate the intensity of vortex through the rotation of velocity, as shown in Eqs (10) ∼ (13).

$$\omega_{x}=\frac{\partial w}{\partial y}-\frac{\partial v}{\partial z}$$ (10)

$$\omega_{y}=\frac{\partial u}{\partial z}-\frac{\partial w}{\partial x}$$ (11)

$$\omega_{z}=\frac{\partial v}{\partial x}-\frac{\partial u}{\partial y}$$ (12)

$$|\omega |=\sqrt{\omega_{x}^{2}+\omega_{y}^{2}+\omega_{z}^{2}}$$ (13)

where, *u*, *v* and *w* are the velocity corresponding to *X*, *Y* and *Z* direction, respectively; *ω_x_*、*ω_y_*、*ω_z_* are vorticity corresponding to *X*, *Y* and *Z* direction, respectively; |*ω*| is the total vorticity.

### *Q* criterion

The Q criterion was proposed by Hunt et al. [29], who split the velocity gradient tensor into symmetric and asymmetric parts (shown in Eqs (14) and (15)). The symmetrical part and asymmetric part represent deformation and rotation, respectively. This method reflects a dynamic balance of deformation and rotation in fluid motion. When *Q* > 0, it means that rotation takes the initiative, that is, the vortex exists.

$$\nabla V=\underset{\text{sym}metric\;\text{term}}{\underset{︸}{\frac{1}{2}(\nabla V+\nabla V^{\text{T}})}}+\underset{\text{asym}metric\;\text{term}}{\underset{︸}{\frac{1}{2}(\nabla V-\nabla V^{\text{T}})}}=S+A$$ (14)

$$Q=\frac{1}{2}({\left\|A\right\|}^{2}-{\left\|S\right\|}^{2})$$ (15)

where, variable S and variable A are tensor of symmetric and asymmetric terms, respectively.

### *λ*_2_ criterion

The *λ*_2_ criterion was proposed by Jeong and Hussain [30] in 1995. He found vortex existed when the symmetric tensor had two negative eigenvalues through solving the symmetric tensor. The eigenvalues of the symmetric tensors are sorted as follows: *λ*_1_> *λ*_2_> *λ*_3_. There must be two negative eigenvalues when *λ*_2_ <0, that is, vortex exists.

### Ω criterion

The Ω criterion was proposed by Liu et al. [31] in 2016. He transformed the asymmetric and asymmetric terms to represent deformation and rotation, respectively. The ratio of the transformed symmetric term to the whole is used to represent the strength of vortex and the corresponding calculation is shown in Eqs (16) and (18). This method dimensionless the threshold range of vortex intensity is limited to 0.5 ∼ 1 [32].

$$\Omega =\frac{b}{a+b+\varepsilon_{\Omega }}$$ (16)

$$a_{\Omega }=\text{trace(}SS^{\text{T}}\text{)}=\overset{3}{\underset{i=1}{\sum }}\overset{3}{\underset{j=1}{\sum }}{\left(S_{ij}\right)}^{2}$$ (17)

$$b_{\Omega }=\text{trace}(AA^{\text{T}}\text{)}=\overset{3}{\underset{i=1}{\sum }}\overset{3}{\underset{j=1}{\sum }}{\left(A_{ij}\right)}^{2}$$ (18)

where, *ε*_Ω_ is the adjustment coefficient. This method has a strong ability to capture weak vortexes for the lower *ε*_Ω_, but it is easy to identify errors. However, some weaker vortex structures will be omitted when the value is too large. Thus, a proper adjustment factor is required, which is 0.01 in the present work.

## Result and discussion

### Vortex identification criteria optimization

**Fig 2:**
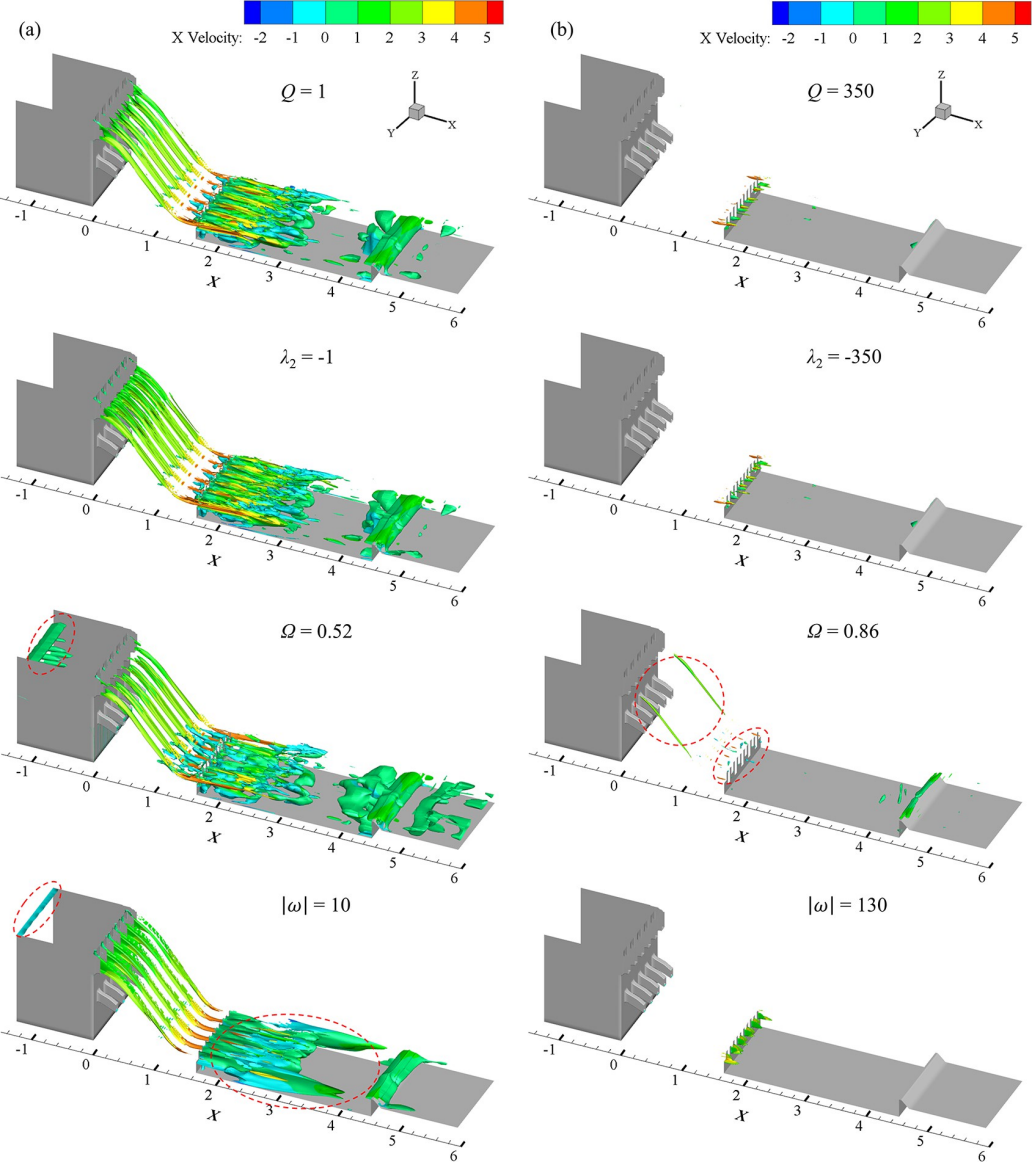
Vortex structure with different vortex identification criteria, (a) small threshold and (b) large threshold.

It is necessary to choose the optimal criteria that are best effective in identifying the vortex in multi-horizontal submerged jets stilling basin before going into a detailed study. Fig 2 shows the recognition results corresponding to different thresholds and different vortex identification criteria in case 1.

As shown in Fig 2A, when the threshold is small, four criteria all can identify the vortex in the stilling basin. A good deal of vortexes is captured in the vicinity of vertical drop for the strong fluctuation. Obvious vortex structures are observed near the inlet of the upstream reservoir area where there is no obvious change of flow pattern when the omega and vorticity criterion are employed. It is believed that there may be slight fluctuation of water that causes the identification error of the vorticity criterion after careful analysis. In addition, there are blank areas appear near the tail ridge within the vorticity criterion. According to the comparative analysis in Fig 2B, the identification effect of Q criterion and *λ*_2_ criterion is almost the same at large threshold values. Obvious vortexes are observed in both crests overflowing orifice and tail ridge of the stilling basin and the coverage of the strength is obviously greater than the vortex near the vertical drop in the stilling basin when Ω criterion is applied. However, vortex with large strength mainly appear in the vicinity of vertical drop. It is considered that the shear deformation and vortex motion of both crests overflowing orifice and tail ridge of the stilling basin are both insignificant, but the ratio of vortex to shear deformation is large and causes the identification error. Although the vorticity criterion can identify the vortex structure near the vertical drop, it is mainly distributed near the center line of the crests overflowing orifice, while the vortexes in other position are not identified.

Easy omission in the process of vorticity criterion to identify smaller vortex intensity, and Ω criterion are easy to identify shear deformation error as a vortex structure. The vortex structure can be well identified by Q criterion and *λ*_2_ criterion, the effect of which are similar. Q criterion is adopted to study the vortex structure in multi-horizontal submerged jets stilling basin in the present work.

### Characteristic of vortex

**Fig 3:**
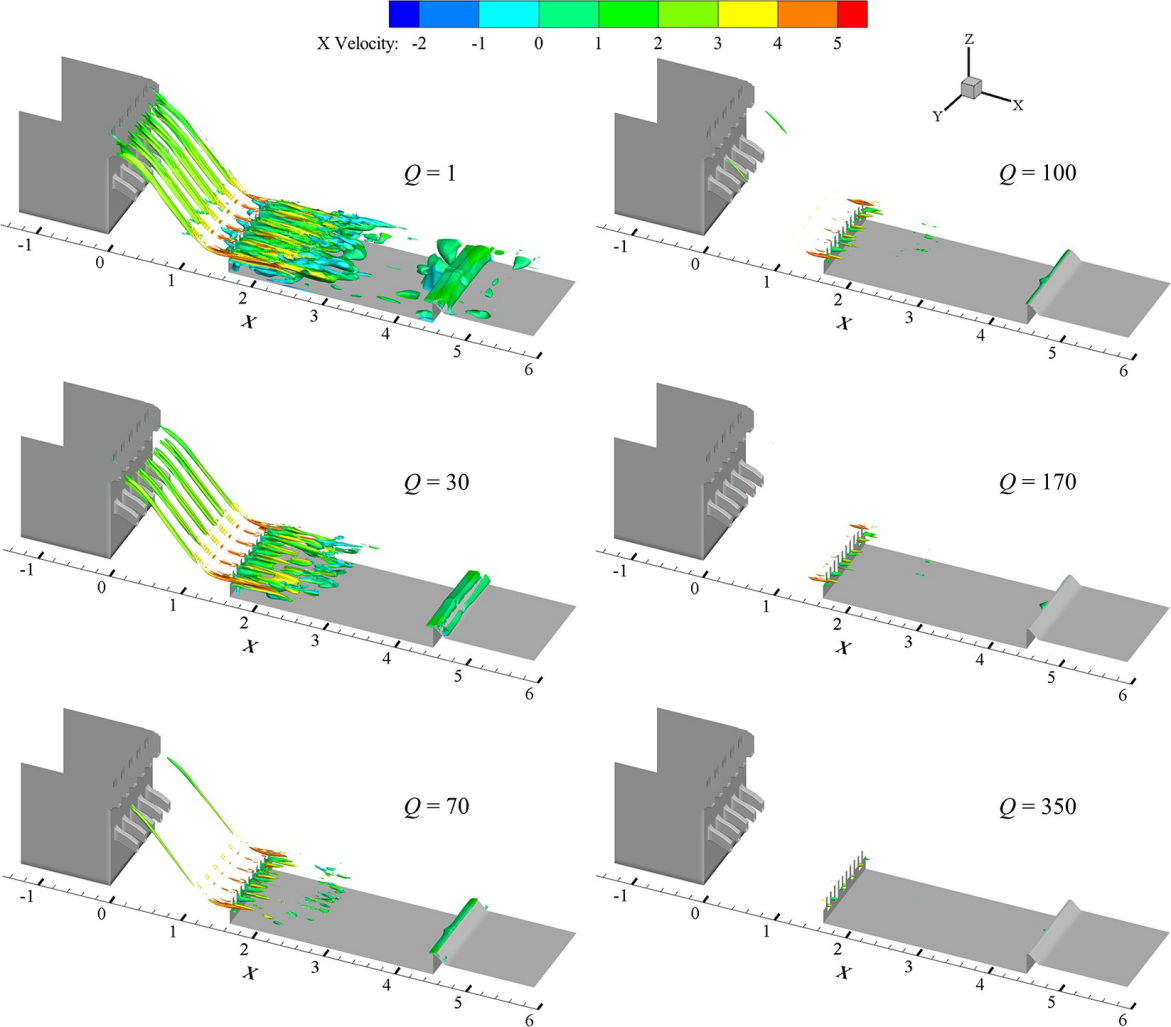
Vortex structure under different thresholds in case1.

The variation law of vortex structure with Q criterion under different threshold values in case 1 is analyzed, as shown in Fig 3.

The vortex intensity increases but the coverage of vortex decreases for the increasing threshold value, according to Fig 3. The vortex intensity identified almost covers the overflowing orifices and upper half of the whole stilling basin (*X* = 0 ∼ 3 m) with *Q* = 1. It can be seen that vortexes with greater intensity are mainly concentrated near the vertical drop (*X* = 1 ∼ 2.8 m) and appear near the COO on both sides and at the tail ridge of the stilling basin, when *Q* = 20. The identified vortex mainly appears in the range of *X* = 1 ∼ 2.6 m and the vortex range gradually decreases with *Q* = 70. When *Q* = 100, The identified vortex structure is also mainly distributed around vertical drop, but the coverage of the vortex is significantly reduced. When *Q* is greater than 100, the coverage of vortex further decreases with a smaller gradient as the *Q* increases, because there are fewer vortexes with this intensity.

**Fig 4:**
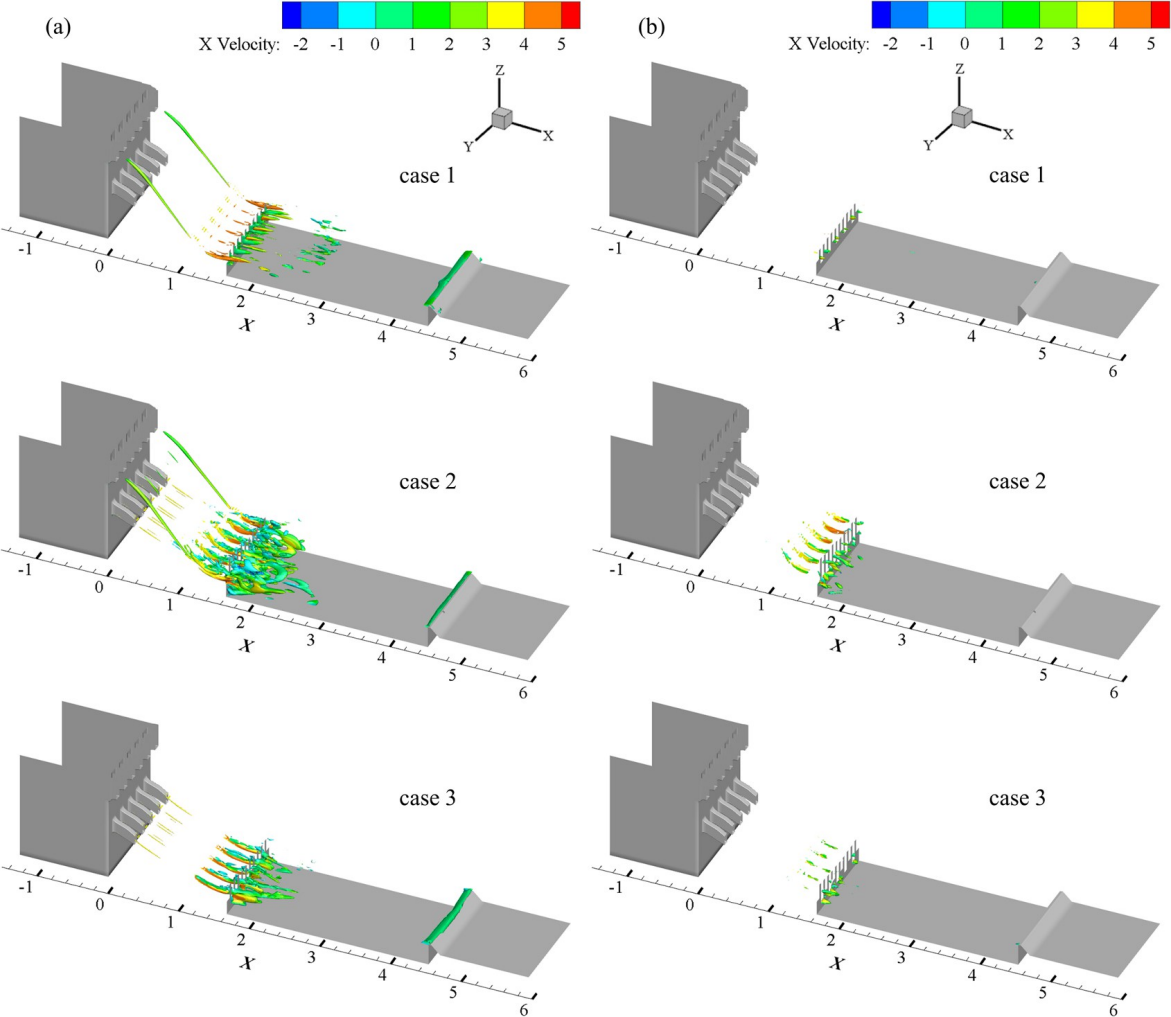
Vortex structures of different aerial drainage with *Q* = 70 (a) and *Q* = 350 (b).

Based on the analysis of the above vortexes, the vortex recognition effects of different flow rate are analyzed under the thresholds *Q* = 70 and *Q* = 350, as shown in Fig 4.

According to Fig 4A, the identified vortex structure can be observed in the COO especially on the two sides in the presence of the COO, but no obvious vortex structure was found in the COO under the condition of the MO when *Q* = 70. In all three cases, obvious vortex structures appear near the vertical drop of the basin and the number of vortex structures identified is the largest under the condition of the COO-MO. The vortex structure of the stilling basin moves a distance to the downstream obviously under the condition of the COO alone. At the same time, obvious vortexes are captured in the tail ridge of the stilling basin in these three cases. As shown in Fig 6B, the strength of the vortex is already quite large, and only a few vortex structures are identified in the stilling basin when *Q* = 300. With this threshold, it is obvious that the number of vortex bodies is relatively large in the case of the COO-MO, while the number of vortex bodies in the case of COO or MO is relatively small and almost invisible. It can be concluded that the combined effect of COO and MO have significant influence on the vortex structure in stilling basin from the analysis of several cases.

**Fig 5:**
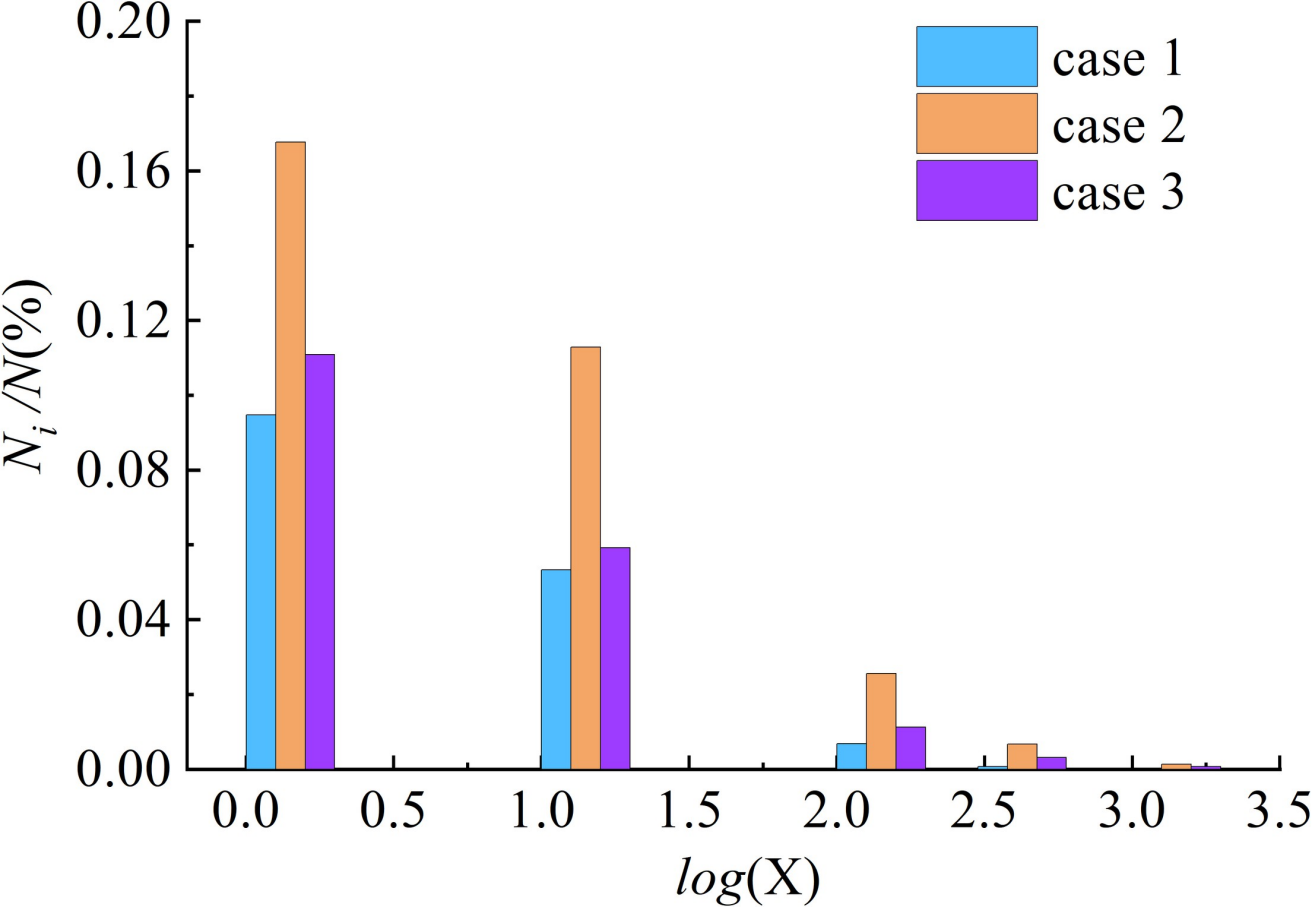
The distribution of vortex with different thresholds.

**Fig 6:**
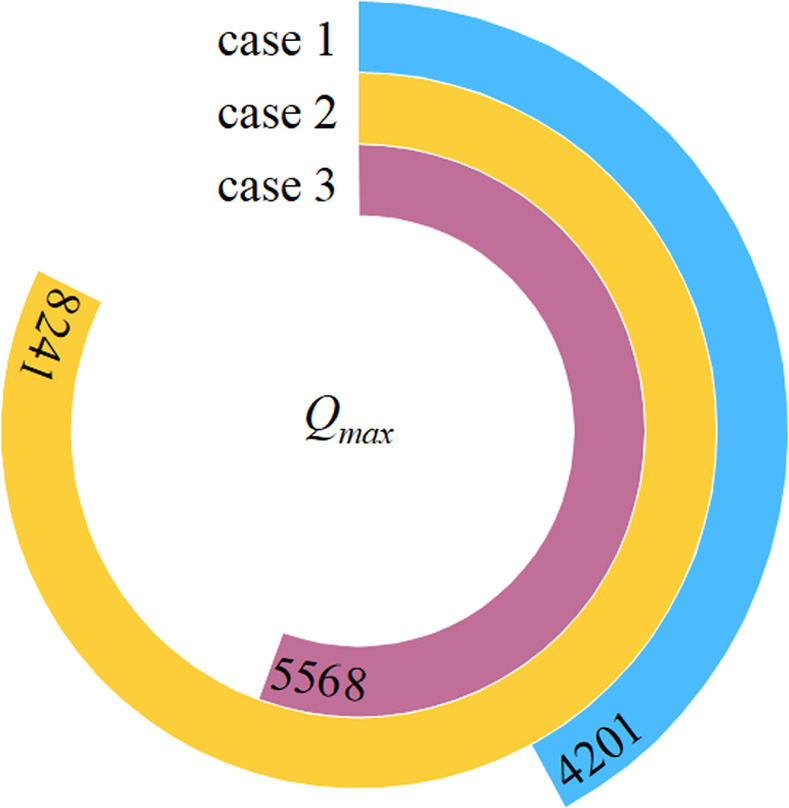
Maximum threshold of vortex in different cases.

The ratio of the number of nodes (*N*_i_) of the vortex structure to all water nodes (*N*) at a certain moment after the final stability is calculated in different thresholds to analyze the distribution law of the vortex structure (shown in Fig 5). The distribution law of maximum threshold in different cases is shown in Fig 6.

As shown in Fig 5, the number of vortices decreases in exponential form with the increase of threshold. For the same threshold, the number of vortices with the MO are larger than that with the COO. The reason is that the coverage of the vortex is relatively large when the flow is discharged from the MO, that is, it exists in both the MO and the stilling pool. However, the vortices with the COO mainly appear in the stilling pool, especially near the entrance of the stilling pool, where the vortices break and reassemble rapidly, accompanied by strong dissipation. The maximum number of vortexes is found with the case of the COO-MO, because the superposition effect occurs, resulting in numerous vortices in the stilling pool and MO. Similarly, the maximum threshold with different aerial drainage modes presents a similar rule (shown in Fig 6). Because the influence of velocity, relative aerial drainage height and aerial drainage mode on the vortex in stilling basin is the result of comprehensive factors.

### Dissipation characteristics of stilling basin

**Fig 7:**
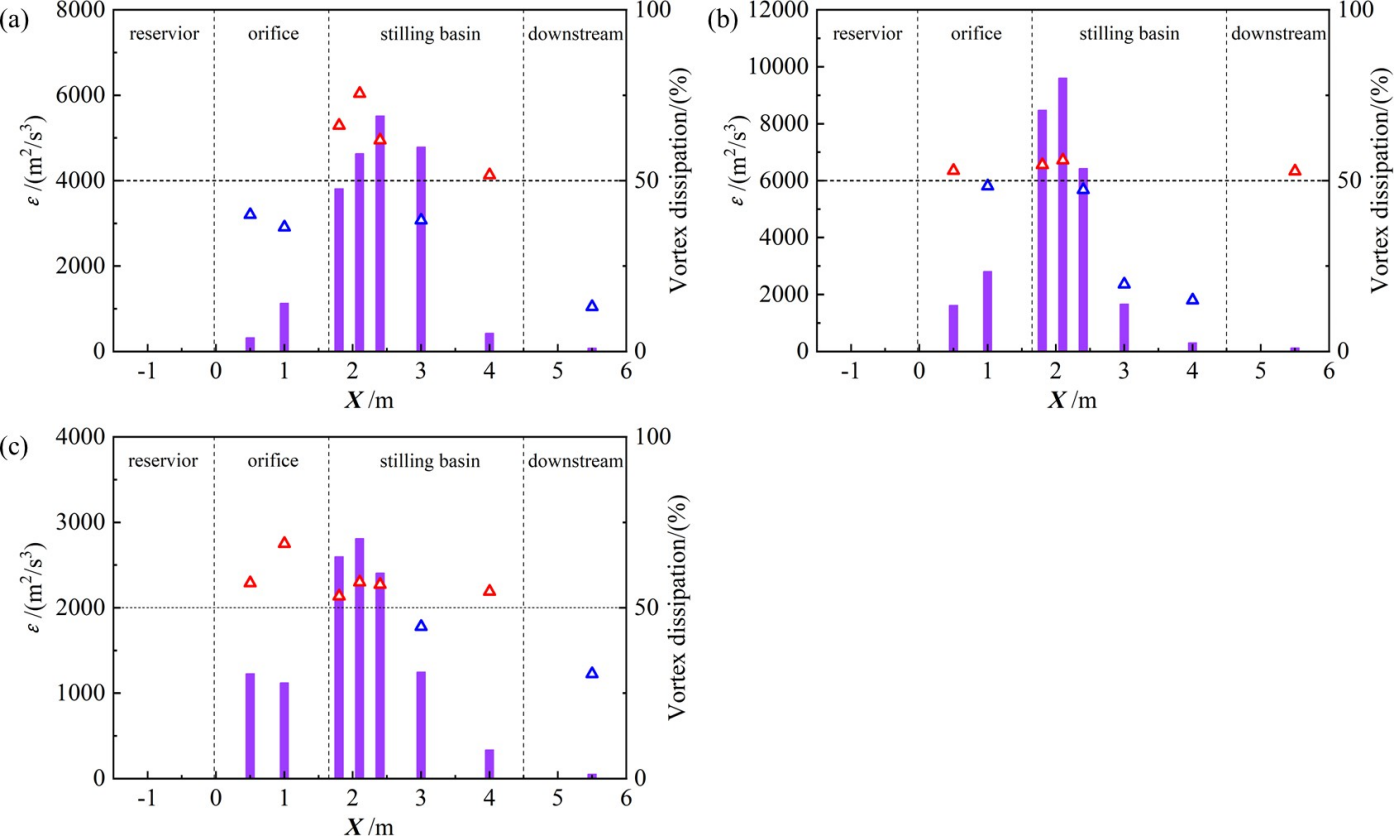
Turbulent dissipation rate in case1 (a), case2 (b), and case3 (c).

A velocity gradient between water and orifice wall leads to shear dissipation. At the same time, vortexes may appear for the change in velocity direction of the water due to the influence of the orifice wall. The velocity in the water deflects obviously due to the sharp change of the shape of the side wall at the vertical drop when the water enters the stilling basin through the orifice. Furthermore, there are obvious vortexes near the vertical drop, which break and merge continuously, and gradually move to the bottom of stilling basin and downstream [4]. The energy of water will expand in these processes. To study the dissipation characteristics, the turbulent dissipation rate of different cases with the scale of 1:80 and 1:200 models are analyzed. According to the definition of Q criterion, the movement of any point in water can be understood as a combination of shear and rotation and it means there exists vortex (rotation plays the main role) when *Q* > 0 but there is no vortex (shear plays the main role) when *Q* < 0. In this work, it is considered that the dissipation rate of nodes is the energy dissipation caused by vortex when *Q* > 0 otherwise is caused by shear. The *Q* values of each node in different sections are counted to the dissipation characteristics, as shown in Fig 7. The left axis represents the dissipation intensity at different locations, and the right axis represents the proportion of vortex dissipation.

As shown in Fig 7, the turbulent dissipation rate increases gradually in the orifice when there exists COO but decreases with the MO alone. It is found that there is a trend of increasing first and then decreasing in the stilling basin section and the maximum turbulent dissipation rate appears in the stilling basin in the vicinity of the vertical drop for a large number of vortexes caused by the dramatical changes of the boundary. The intensity and coverage of the vortexes are getting weaker from the vicinity of the vertical drop to the downstream, causing the dissipation rate of turbulence decreases. In the outlet section, the turbulent dissipation rate, closing to zero, is much smaller than that of the vertical drop for that the flow is relatively stable.

With further analysis of the turbulent dissipation rate, it is found that the direction of velocity is deflected for the abrupt change of the inlet of the orifice, causing that the vortex dissipation is stronger than the shear dissipation. The intensity of vortex becomes weaker but the velocity gradient at the orifice wall of orifice becomes larger causing that shear dissipation is greater than vortex dissipation from inlet to downstream. However, the vortex dissipation still plays the main role with the MO for the small orifice height and velocity gradient. In the stilling basin, there are many violent vortexes near the vertical drop, where the dissipation of vortexes plays main role especially with MO. The coverage of vortex influence is getting smaller, and the effect of shear dissipation is gradually increase from the vicinity of the fall to the downstream, thus the difference between them is not significant. However, the shear dissipation at the downstream of stilling basin is better than that of vortex dissipation in the downstream of the stilling basin for the large velocity gradient with the COO-MO. In the exit section, the dissipation rate, closing to zero, is greatly constituted of shear dissipation.

## Conclusion

Four vortex identified criteria are adopted to analyze the characteristics of vortex and dissipation in the multi-horizontal submerged jets stilling basin with different aerial drainage modes. The following conclusions are drawn:

1. Among the four vortex identified criteria, Q criterion and *λ*_2_ criterion have a good effect on the identification of vortex structure in the multi-horizontal submerged jets stilling basin studied in this paper. Easy omission in the process of vorticity criterion to identify smaller vortex intensity, and Ω criterion are easy to identify shear deformation error as a vortex structure.
2. The way of aerial drainage has a significant impact on the vortex structure in the stilling basin and the intensity and number of vortexes with COO-MO are the largest.
3. The energy dissipation of water in the orifice are mainly reflected by shear dissipation. The vortex dissipation is the main role in the main vortex area of the stilling basin near vertical drop, although the shear dissipation is still large.

In this work, the evolution and distribution characteristics of vortices in Multi-horizontal Submerged Jets Stilling Basin are studied by numerical simulation and the results can provide guidance for the reinforcement of floor and side wall in the actual stilling pool.

## Supporting information

S1 File Vortices with different thresholds. (XLSX)

S2 File Maximum threshold in different cases. (XLSX)

S3 File Turbulent dissipation rate in different cases. (XLSX)

S1 Data (XLSX)

## References

[1] Zhang JM., Chen JG. An energy dissipator with multi-horizontal submerged jets. Chengdu: Science press, 2013.

[2] Deng J, Xu WL, Zhang JM, Qu JX, Yang YQ. A new type of plunge pool—multi-horizontal submerged jets. Sci China Ser E-Technol Sci. 2008; 51(12):2128–2141. doi: 10.1007/s11431-008-0237-z

[3] Chen JG, Zhang JM, Xu WL, Wang YR. Numerical simulation of the energy dissipation characteristics in stilling basin of multi-horizontal submerged jets. J Hydrodyn. 2010; 22(5):732–741. doi: 10.1016/S1001-6058(09)60110-4

[4] Chen JG. Theoretical study and numerical simulation on the multi-horizontal submerged jets. Sichuan University, 2012.

[5] Chen JG, Zhang JM, Xu WL, Peng Y. Characteristics of the velocity distribution in a hydraulic jump stilling basin with five parallel offset jets in a twin-layer configuration. J Hydraul Eng. 2014; 140(2):208–217. doi: 10.1061/(ASCE)HY.1943-7900.0000817

[6] Peng Y, Zhang JM, Xu WL, Rubinato M. Experimental optimization of gate-opening modes to minimize near-field vibrations in hydropower stations. Water. 2018; 10(10):1435. doi: 10.3390/w10101435

[7] Zhang JM., Wang YR., Yang YQ, Xu WL, Li YL, Zeng XH, et al. Energy dissipation and hydraulics characteristics of multi-horizontal submerged jets. Adv Water Sci. 2005,16(1):18–22 (in Chinese). http://skxjz.nhri.cn/en/article/id/1010.

[8] Li YL., Yang YQ. Hua GC. et al. Experimental study on multi-horizontal submerged jets. Journal of Sichuan University. 2004; 36(6):32–36.

[9] Chen JG, Zhang JM, Xu WL, Wang YR. Scale effects of air-water flows in stilling basin of multi-horizontal submerged jets. J Hydrodyn. 2010; 22(6):788–795. doi: 10.1016/S1001-6058(09)60117-7

[10] Moradi M, Sajjadi M, Balachandar R, Arman A, Ilinca A. Experimental analysis of multi-horizontal submerged jets energy dissipater. ISH J. Hydraul. 2022; 28(3):281–291. doi: 10.1080/09715010.2021.1885508

[11] Zhao Z, Wang JX, Zhu DZ. Energy dissipation in a deep tailwater stilling basin with partial flaring gate piers. Can. J. Civ. Eng. 2020; 47(5): 523–533. doi: 10.1139/cjce-2018-0099

[12] Li S, Yang J, Ma X, Li X. Flow features in a pooled fishway with V-shaped weir formation. ENG. APPL. COMP. FLUID,2020; 14(1): 1337–1350. doi: 10.1080/19942060.2020.1829710

[13] Gao P. Yang Y. Q. Deng J. et al. Investigation on Complex flow pattern of multi-submerged jets into plunge pool [J]. Journal of Sichuan University. 2006; 38(5):70–75. https://jsuese.scu.edu.cn/jsuesecn/ch/reader/viewabstract.aspx?flag=1&fileno=20060511&journalid=jsuesecn.

[14] Karimpour A, Kaye NB, Khan AA. CFD study of merging turbulent plane jets. J Hydraul Eng. 2011; 137(3):381–385. doi: 10.1061/(ASCE)HY.1943-7900.0000308

[15] Lai JCS, Nasr A. Two parallel plane jets: Comparison of the performance of three turbulence models. Proc Inst Mech Eng Part G J Aerosp Eng. 1998; 212(6):379–391. doi: 10.1243/0954410981532351

[16] Mao YF, Guan MF. Mesh-free simulation of height and energy transfer of landslide-induced tsunami waves. Ocean Eng. 2023; 284: 115219. doi: 10.1016/j.oceaneng.2023.115219

[17] Mao YF, Kong Y, Guan MF. GPU-accelerated SPH modeling of flow-driven sediment erosion with different rheological models and yielcriteria. Powder Technol. 2022; 412: 118015. doi: 10.1016/j.powtec.2022.118015

[18] Yang Z. C., Deng J., Yang Y. Q., et al. Numerical simulation of multiple submerged jets on multilevel discharged into plunge pool. J Hydraul. 2004; (5): 31–38 (in Chinese). http://jhe.ches.org.cn/jhe/ch/reader/viewabstract.aspx?file_no=2004050031&flag=1.

[19] Deng J. Xu WL. Zhang JM. et al. Test and numerical simulation of the plunge pool for Xiangjiaba dam. Water Power. 2004; 30(11):12–15. (in Chinese).

[20] Chen JG, Zhang JM Xu WL, Li S, He XL. Particle image velocimetry measurements of vortex structures in stilling basin of multi-horizontal submerged jets. J Hydrodyn. 2013; 25(4):556–563. 10.1016/S1001-6058(11)60396-0.

[21] Zhang JM, Chen JG, Xu WL, Peng Y. Characteristics of vortex structure in multi-horizontal submerged jets stilling basin. P I Civil Eng-WAT M. 2014; 167(6):322–333. doi: 10.1680/wama.12.00071

[22] Tian SL, Gao YS, Dong XR, Liu CQ. Definitions of vortex vector and vortex. J Fluid Mech. 2018; 849:312–339. doi: 10.1017/jfm.2018.406

[23] Zhang YN, Qiu X, Chen FP, Liu KH, Dong XR, Liu CQ. A selected review of vortex identification methods with applications. J Hydrodyn. 2018; 30(5):767–779. doi: 10.1007/s42241-018-0112-8

[24] Yakhot V, Orszag SA. Renormalization-group analysis of turbulence. I. Basic theory. J Sci Comput. 1986; 1:3–11. doi: 10.1007/BF01061452 10033528

[25] Han Z, Reitz RD. Turbulence modeling of internal combustion engines using RNG κ-ε models. Combust Sci Technol. 1995; 106(4):267–295. doi: 10.1080/00102209508907782

[26] Hirt CW, Nichols BD. Volume of fluid (VOF) method for the dynamics of free boundaries. J Comput Phys. 1981; 39(1):201–225. doi: 10.1016/0021-9991(81)90145-5

[27] Issa RI. Solution of the implicitly discretised fluid flow equations by operator-splitting. J Comput Phys. 1986; 62(1):40–65. doi: 10.1016/0021-9991(86)90099-9

[28] Metcalfe R. M., Menon S., Hussain A. K. M. F. Coherent structures in a turbulent mixing layer a comparison between numerical simulations and experiment. Turbulent Shear Flows, 1985, 5: 110. 10.1007/978-3-642-71435-111.

[29] Hunt J. C. R., Wray A. A., Moin P. Eddies, streams, and convergence zones in turbulent flows. Studying Turbulence Using Numerical Simulation Databases, San Francisco, USA,1988: 193–208.

[30] Jeong J, Hussain F. On the identification of a vortex. J Fluid Mech. 1995; 285:69–94. 10.1017/S0022112095000462.

[31] Liu CQ, Wang YQ, Yang Y, Duan ZW. New omega vortex identification method. Sci China Phys Mech Astron. 2016; 59(8): 684711. doi: 10.1007/s11433-016-0022-6

[32] Liu CQ, Gao YS, Dong XR, Wang YQ, Liu JM, Zhang YN, et al. Third generation of vortex identification methods: Omega and Liutex/Rortex based systems. J Hydrodyn. 2019; 31(2):205–223. 10.1007/s42241-019-0022-4.

